# Adjunctive subgingival application of Chlorhexidine gel in nonsurgical periodontal treatment for chronic periodontitis: a systematic review and meta-analysis

**DOI:** 10.1186/s12903-020-1021-0

**Published:** 2020-01-31

**Authors:** Han Zhao, Jingchao Hu, Li Zhao

**Affiliations:** 1 Multi-disciplinary Treatment Center, Beijing Stomatological Hospital, School of Stomatology, Capital Medical University, Tian Tan Xi Li Number.4, Beijing, 100050 China; 2 Department of Periodontology, Witten/Herdecke, University, Alfred-Herrhausen-Str. 45, 58445 Witten, Germany; 3 Department of Periodontics, Beijing Stomatological Hospital, School of Stomatology, Capital Medical University, Tian Tan Xi Li Number 4, Beijing, 100050 China; 4 Department of Prosthodontics, Stomatological Hospital of Chongqing Medical University, Chongqing, 400015 China; 5 Chongqing Key Laboratory of Oral Diseases and Biomedical Sciences, Chongqing, 400015 China; 6 Chongqing Municipal Key Laboratory of Oral Biomedical Engineering of Higher Education, Chongqing, 400015 China

**Keywords:** Chronic periodontitis, Chlorhexidine, Subgingival irrigation, Root planing; meta-analysis

## Abstract

**Background:**

Subgingival applications of chlorhexidine (CHX) gel are commonly used as an adjunct in nonsurgical periodontal treatment (NSPT) for chronic periodontitis (CP). However, there is lack of systematic review and meta-analysis justifying the effects of adjunctive CHX gel on clinical outcomes. The objective of this meta-analysis was to evaluate the efficacy of adjunctive subgingival administration of CHX gel in NSPT compared to NSPT alone for CP.

**Methods:**

An electronic search of four databases and a manual search of four journals were conducted up to August 2019. Only randomized controlled trials reporting on the clinical outcomes of subgingival use of CHX gel adjunct to scaling and root planing (SRP), as compared to SRP alone or with placebo, for at least 3 months were included. Primary outcomes were probing pocket depth (PPD) reduction and clinical attachment level (CAL) gain at 3 and 6 months, when data on at least three studies were obtained.

**Results:**

Seventeen studies were included for qualitative analysis and seven studies for quantitative analysis (four studies for the application of CHX gel adjunct to SRP at selected sites with at least pocket depth ≥ 4 mm and three studies for comparison of full-mouth disinfection (FMD) with subgingival use of CHX gel and full-mouth scaling and root planing (FMSRP). For subgroups, the clinical outcomes between adjunctive use of Xanthan-based CHX gel (XAN-CHX gel) and CHX gel were analyzed. Results indicated a significant improvement of PPD reduction following local adjunctive administration of XAN-CHX gel for SRP at selected sites (MD: 0.15 mm). However, no difference was found in CAL gain. Moreover, no significant difference was observed in PPD and CAL at both 3 and 6 months post-treatment between FMD and FMSRP.

**Conclusion:**

Adjunctive subgingival administration of XAN-CHX gel at individual selected sites in NSPT appears to provide slight benefits in PPD reduction compared to NSPT alone for CP. Due to the lack of high-quality studies, further studies with larger sample sizes and strict standards are needed to confirm the conclusions.

## Background

Chronic periodontitis (CP) is characterized as a complex progressive chronic inflammatory process, which leads to the destruction of periodontal supportive tissue and a further loss of teeth. CP occurs when the magnitude effects of the pathogenic microbial load in the periodontal pocket are larger than that of the hosts immune response [1, 2]. The basis of periodontal treatment is elimination or suppression of periodontal pathogens. The golden standard of which is mechanical debridement by scaling and root planing (SRP). However, large limitations of physical treatment have been observed due to the difficulty of accessing deep periodontal defects, which compromises the effectiveness of biofilm removal. The persistence of periodontal pathogens, such as *Aggregatibacteractinomycetemcomitans* and *Porphyromonas gingivalis* (P.g), were often found following SRP and can result in microbial re-colonization and the consequent destruction of periodontal tissue [3–6]. In regards to this issue, adjunctive systemic and localized antibiotics have been applied to compensate for the limitation of mechanical therapy. Despite the rapid development of a variety of adjunctive local periodontal treatments in recent years, such as metformin, antioxidants, photodynamic treatment and so on [7–9], chlorhexidine (CHX) remains one of the most effective local antimicrobial agents, and is widely used for the local treatment of periodontitis [10–13]. Through the rapid attraction of the negatively charged bacterial cell surface to the cationic CHX molecule, CHX shows strong antibacterial activity in the periodontal pocket, along with a lack of toxicity, incompliance from patients and an emergence of resistance microorganisms. However, the high clearance of CHX from the periodontal pocket leads to subtherapeutic CHX concentrations in the local environment after only a short time of subgingival CHX application [14], which results in an insufficient treatment effectiveness [1, 15]. Given this limitation, CHX Gel with CHX concentration up to 15 times than liquid carriers was developed for periodontal treatment. In recent years, numerous of studies have reported the effectiveness of adjunctive CHX to nonsurgical periodontal treatment (NSPT). However, contrary results were presented [10–13], there is still no consensus on this issue. So far, only one systematic review without quantitative analysis indicated that the positive effect of local subgingival application of CHX Gel adjunctive to NSPT could be not justified as compared to NSPT alone [16]. Therefore, there is lack of strong evidence for support the beneficial effect of subgingival use CHX as adjunct to NSPT.

Full-mouth disinfection (FMD) was proposed by Quirynen in 1995, with the aim of eradicating periodontal pathogens in a short time from all the oropharyngeal habitats (mucous membranes, tongue, tonsils and saliva) [17]. CHX gel as an adjunct was used in the FMD protocol, which was described as full-mouth scaling and root planing (FMSRP) in 1–2 sessions within 24 h combined with full-mouth subgingival irrigation with CHX gel, as well as a tongue brush and mouthwash by means of CHX [2, 17–20]. However, whether the use of antiseptics played a role in FMD is still unclear.

The aim of this systematic review and meta-analysis was to evaluate the benefits of a subgingival administration of CHX gel as an adjunct to NSPT for the treatment of CP.

## Method

### Focus questions

Whether subgingival chlorhexidine gel application as an adjunct to nonsurgical periodontal treatment provides additional benefit to clinical outcomes in chronic periodontitis?

### Search strategy

The review and meta-analysis were based on the Preferred Reporting Items for Meta-Analysis (PRISMA) statement [21]. Three reviewers (HZ, JCH and LZ) conducted an independent search of three databases, including PubMed, EMBASE and the Cochrane Collaboration Library on the 20 August 2019 for articles addressing the focused question. Furthermore, a search of the Open Grey database was performed, and a hand search was conducted of following journals: Journal of Dental Research, Journal of Periodontology, Journal of Clinical Periodontology and Journal of Periodontal Research from 2000 until 2019.

### Study selection

Titles and abstracts were reviewed for eligibility by two independent reviewers (HZ, JCH) according to the inclusion criteria. Studies that met all inclusion criteria or met some of inclusion criteria but did not meet any of the exclusion criteria were admitted for full-text review. In this phase, full-text papers were assessed in line with the exclusion criteria. And the reasons for exclusion were recorded (Additional file 1: Table S1). Any disagreements were resolved on discussion between the three reviewers and a consensus was reached through voting. The agreement value between the reviewers was calculated using Kappa statistics, which is used to measure inter-rater reliability. The classification of Kappa Value was suggested: ≤ 0 as indicating no agreement, 0.01–0.20 as none to slight, 0.21–0.40 as fair, 0.41–0.60 as moderate, 0.61–0.80 as substantial, and 0.81–1.00 as perfect agreement [22].

The search strategy for PubMed (adapted to the other databases) is listed below:

(periodontitis OR periodontal disease) AND ((((chlorhexidine, OR chlorhexidine gluconate, OR xanthan OR xanthan chlorhexidine) AND gel) AND (subgingival, OR subgingival curettage, OR dental scaling, OR root planing OR dental prophylaxis)) OR full mouth disinfection)

### Primary and secondary outcomes

The primary outcomes were probing pocket depth (PPD) reduction and the clinical attachment level (CAL) gain at 3 and 6 months post-therapy. The secondary outcome was adverse events.

### Eligibility criteria

The inclusion criteria for the studies were: 1) randomized controlled trials (RCTs); 2) comparison of SRP alone/placebo and CHX gel adjunct to SRP; or comparison of FMSRP alone/placebo and FMD, including subgingival use of CHX gel; 3) follow-up of at least 3 months; 4) reported data on clinical parameters (CAL and PPD) and 5) publication in English only.

The exclusion criteria were: 1) not RCTs; 2) duplicate publications; 3) Inadequate treatment strategy: CHX was used as mono-therapy or CHX was used as adjunct to surgery or other treatment; 4) follow-up less than 3 months; 5) reported only microbiological findings with no reference to clinical result; 6) not in English.

### Quality assessment

The methodology quality of the included articles was evaluated independently by two reviewers (HZ and JCH) based on recommendations from the CONSORT statement [23]. Quality assessments of the included studies were conducted using the revised risk of bias assessment tool from the Cochrane Collaboration’s handbook version 5.2.0 [24], which includes seven criteria: random sequence generation; allocation concealment; blinding of participants and personnel; blinding of outcome assessment; incomplete outcome data; selective reporting and other sources of bias. Each category was estimated on whether it could impact the overall results and was further qualified as either low, high or unclear. Overall, each article was judged as (i) low risk of bias, (ii) unclear risk of bias or (iii) high risk of bias. When a trial did not meet all four criteria for randomization and blinding methods, it was excluded from quantitative analysis, as its low quality and high bias may have subverted the validity of the results and conclusions.

### Data collection process/data items

Data of each included study were recorded using a standardized data extraction form, including study design, number of patients, demographics, inclusion criteria, types of CHX gel, timing and frequency of CHX gel application, number of adverse events and length of follow-up.

### Data synthesis

The meta-analysis was performed using RevMan version 5.3 (2014). Mean differences (MD) with 95% confidence intervals (95% CI) were used for continuous data. The I2 value was used to access the statistical heterogeneity of the studies. If the heterogeneity was evaluated as I2 ≤ 50%, a fixed effects model was applied. When the heterogeneity was assessed as I2 > 50%, a random effects model was used. The inverse-variance method performed, and the overall effect was defined as statistically significant if the *p* value < 0.05.The I2 value was classified into four levels: i) no heterogeneity, between 0 and 25%; ii) low heterogeneity, 25–50%; iii) moderate heterogeneity, 50–75% and iiii) high heterogeneity, 75–100% [25].

## Results

### Study selection

**Fig. 1:**
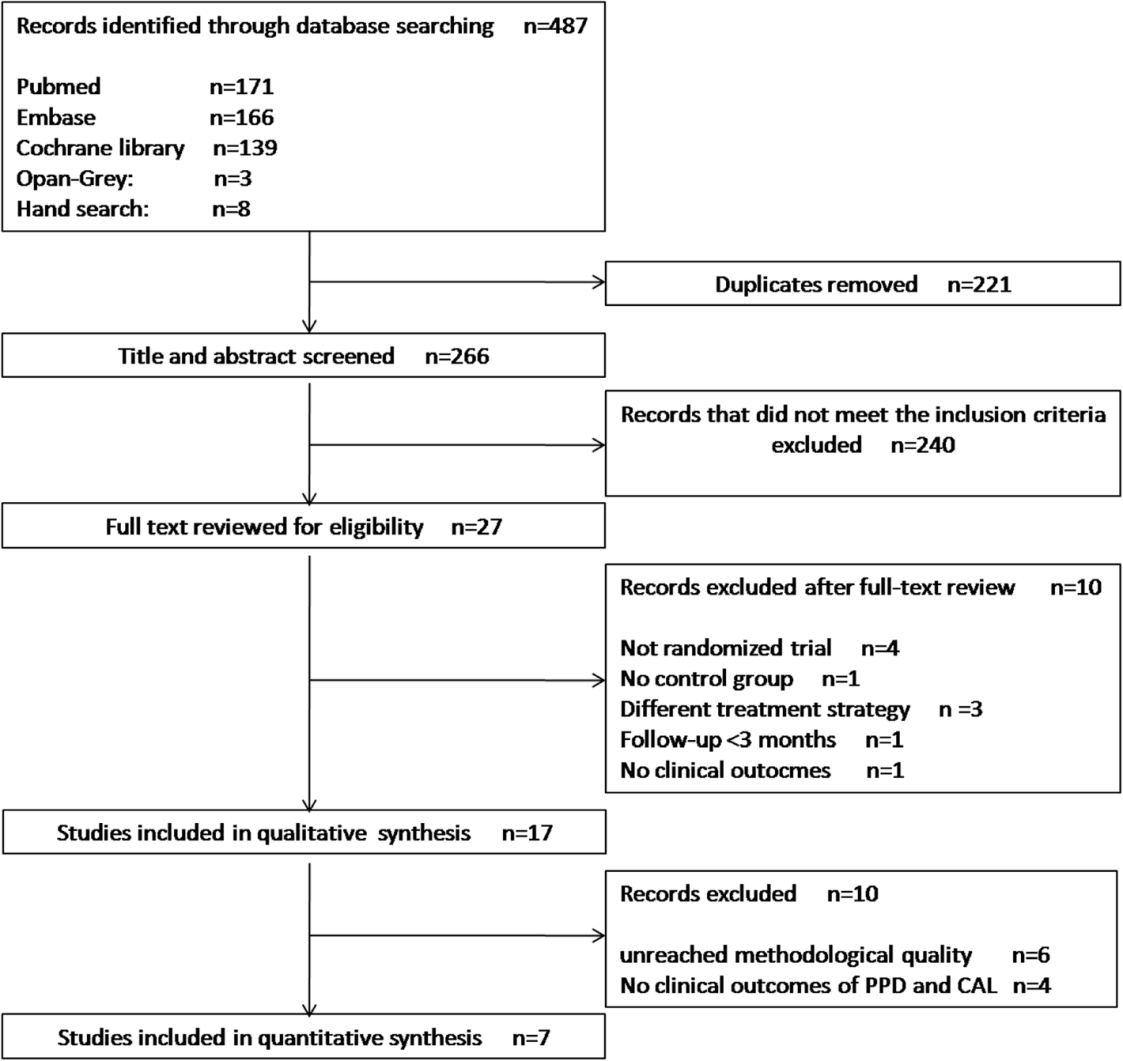
Flow chart of the study identification based PRISMA19 with the reasons for exclusion

In the initial search, a total of 487 studies were identified; 171 PubMed, 166 Embase and 139 from the Cochrane Library database. Three papers were found through the Open Grey search and eight papers were selected following a manual search. After removal of duplicates (*n* = 221), 266 papers were included in the selection phase of titles and abstracts. A total of 240 articles were excluded, and 27 papers were selected for full-text reading. In this phase, 10 studies were further excluded (Additional file 1: Table S1) and 17 papers were finally included in the qualitative analysis [2, 10–12, 17–19, 26–35]., The kappa value for inter-reviewer agreement was 0.92 indicating high degree of inter-rater reliability. Figure 1 shows the study identification flowchart based PRISMA19 with the reasons for exclusion.

### Description of the included studies

Seventeen articles met the criteria and were included for qualitative analysis. Thirteen studies reported subgingival application of CHX gel adjunct to SRP at selected sites with a moderate to deep probing depth (at least 4 mm in all studies) [8–10, 25–34]. Nine studies were split-mouth RCTs [10, 12, 27–29, 32–35], four were parallel RCTs [11, 26, 30, 31] and three studies used a placebo in the control [12, 31, 33]. From the 13 papers, 10 showed the clinical outcomes of adjunctive subgingival delivered Xanthan-based CHX gel (XAN-CHX gel) in SRP and SRP alone [26–35]; CHX concentration in the XAN-CHX gel was 1.5% in nine studies and 2.5% in one study [34], and another three studies reported the use of gels containing 0.5, 1 and 2% CHX without Xanthan gum [10–12]. Patient samples ranged from five to 98. One included study compared the clinical outcomes between SRP plus XAN-CHX gel and SRP alone for patients with diabetes mellitus type 2 [27]. The timing and frequency of CHX Gel application varied between the trials. In all 13 studies but four [10, 12, 27, 32], the CHX gel was applied once at baseline after SRP. In the other four studies, the application of CHX gel was described as three times at baseline, 10 day and 20 day follow-ups [27], once at 1 month after treatment [31] and three times within 10 min at baseline [10, 12]. The follow-ups ranged from 1 month to 6 months after SRP.

An additional arm of the four studies evaluated the results between FMD and FMSRP [2, 17–19]. All studies were RCTs, and one used a placebo gel and solution in the FMSRP group. The number of participants ranged from 18 to 38. Follow-ups varied from 1 month to 12 months. One study included patients with diabetes mellitus type 2 [18]. A 1% CHX gel was used in all of the trials. The timing and frequency variations for the CHX gel ranged from once at baseline [17, 19] and three times in 10 min at baseline [18] to three times within 10 min at first session, second session of FMSRP and at 1 week of follow-up, respectively [2]. Table 1 shows the summary of the characteristics of the included studies.

**Table 1:** Characteristics of included studies. Variables were listed in this systematic review (including:study design, patient demographics, methodology, number of adverse events and length of follow-up). Outcome difference is reported only between adjunctive CHX gel to SRP and SRP alone

Administration	Study	Design	Participants				Methodology		AE	Follow-up (m)
			N (C/T)	Inclusion criteria	SD	age	Description of Gel	CHX Gel Application		
Application at selected sites	Faramarzi M et al. (2017) [26]	P	68 (34/34)	at least eight teeth with PD 4-8 mm	2-DM	30–60 years	XAN-CHX 1.5% CHX gel	one time after 2nd SRP (baseline, 2 week after 1st SRP)	/	3,6
	Phogat M et al. (2014) [27]	S	30 (30/30)	at least 3 nonadjacent interproximal sites with PD 4-8 mm	no	30–50 years	XAN-CHX 1.5% CHX gel	one time at baseline, 10 days and 20 days	/	1,3
	Jain M et al. (2013) [28]	S	30 (30/30)	2 sites located on the same side PD between 5 to 7 mm	no	30–60 years	XAN-CHX 1.5% CHX gel	one time at baseline	/	1.5,3,6
	Chitsazi MT et al. (2013) [29]	S	20 (20/20)	one site per quadrant with PD ≥4 mm and BOP (+)	no	mean 46.5 years	XAN-CHX 1.5% CHX gel	one time at baseline	0	1,3
	Chauhan AS et al. (2013) [30]	P	40 (20/20)	at least 8 teeth with PD 4-8 mm	no	30–65 years	XAN-CHX 1.5% CHX gel	one time at baseline	/	1,3
	Matesanz P et al. (2013) [31]	P (placebo)	22 (12/10)	at least 16 teeth and at least 3 teeth per quadrant,4–10 pockets with PPD > 4 mm and BOP(+), or at a programmed supportive visit	no	elder than 30 years	XAN-CHX 1.5% CHX gel	one time at baseline	0	1,3,6
	Verma A et al. (2012) [32]	S	46 (46/46)	at least two non-adjacent interproximal sites with PD5-8 mm and BOP(+)	no	30–65 years	XAN-CHX 1.5% CHX gel	one time 1 month after SRP	/	1,3
	Kranti K et al. (2010) [33]	S (placebo)	10 (10/10)	at least 4 periodontal pockets with PPD 5-8 mm	yes	25–65 ears	XAN-CHX 1.5% CHX gel	one time at baseline	/	3,6
	Paolantonio M (2009) [34]	S	98 (98/98)	at least two teeth with PD ≥5 mm and BOP (+)	no	24–58 years	XAN-CHX 2.5% CHX gel	one time at baseline	/	3,6
	Gupta R et al. (2008) [35]	S	30 (30/30)	at least three teeth, (at least one tooth apart), with PPD 5-8 mm and BOP (+)	no	25–75 years	XAN-CHX 1.5% CHX gel	one time at baseline	/	1,3
	lecic J et al. (2016) [10]	S	5 (5/5)	at least two bilateral PPD ≥ 5 mm	no	21–52 years	0.5% CHX gel	three times within 10 min	/	1,3
	unsal E et al. (1994) 11	P	15 (8/7)	at least 3 teeth in each quadrant with 2 sites with PPD ≥ 4 mm and BOP(+)	no	30–57 years	1% CHX gel	One time at baseline	/	3
	Oosterwaal PJM et al. (1991) [12]	S (placebo)	10 (10/10)	at least 4 interdental PPD 7-9 mm in single rooted teeth and BOP(+)	no	33–62 years	2% CHX gel	3 times within 10 min at baseline	/	1,3,6
Full-mouth application	Fonseca DC et al. (2015) [17]	P	30 (15/15)	mild to moderate chronic periodontitis, at least 18 natural teeth	yes	35–60 years	1% CHX gel	one time at baseline	/	3,6
	Santos VR et al. (2013) [18]	P (placebo)	37 (18/19)	at least 15 teeth, 30% of the sites with concomitant PD and CAL > 4 mm	2-DM	37–75 years	1% CHX gel	3 times within 10 min at baseline	T:17 C:12	3,6,12
	swierkot et al. (2009) [19]	P	18 (9/9)	at least 20 teeth with at least six sites PPD ≥5 mm and BOP(+)	no	28–63 years	1% CHX gel	one time at baseline	0	1,2,4,8
	Quirynen M et al. (2006) [2]	P	28 (14/14)	at least 18 teeth, at least 2 multi-rooted and/or 2 single-rooted teeth in the first quadrant, at least 6 sites PPD 6 mm, radiographic bone loss≥25%	no	30–75 years	1% CHX gel	three times within 10 min at first sessecion, second session, and 1-week follow-up	/	2,4,8

Studies varied according to the design type of studies, the inclusion or exclusion of patients with systemic disease, different concentration and composition of chlorhexidine gel and different timing and frequency of CHX gel application. Adverse events and follow-up period were recorded

*P* Intersubject parallel study, *S* Intrasubject split-mouth study, *N* Number, *T* Test group, *C* Control group, *SD* Systemic disease, *2-DM* Diabetes mellitus type 2, *XAN* Xanthan gum, *CHX* Chlorhexidine, *XAN-CHX* Xanthan-based chlorhexidine, *min* Minutes, *AE* Adverse events, *m* Month/months

### Risk of bias assessment

All studies were RCTs. Seven studies did not report on their randomization and allocation methods in detail [11, 12, 27, 28, 30, 32, 34, 35], and from these, six studies also did not describe the blinding methods of participants and personnel as well as their assessment [11, 27, 28, 30, 32, 35], continued CHX rinsing stains the tooth and tongue surfaces, examiners could deduce which subjects were receiving CHX though these changes, and all the four studies in the analysis for comparison between FMD and FMSRP were considered at most to be single-blinded^2,1719^. Given examiner blinding was performed strictly in three studies, the detection bias for the three articles was qualified as ‘unclear’ [2, 17, 19]. Overall, for all 17studies, six were assessed to have a low risk of bias [10, 17, 18, 26, 29, 31], two were judged as an unclear risk of bias [12, 33], nine were considered to have a high risk of bias [2, 11, 19, 27, 28, 30, 32, 34, 35], and six were excluded from the quantitative analysis [11, 27, 28, 30, 32, 35]. . The summary of quality assessment is showed in Table 2.

**Table 2:** Risk of bias assessment

Author (year)	Random sequence generation	Allocation concealment	Blinding of participants and personnel	Blinding of outcome assessment	Incomplete outcome data	Selective reporting	Other bias	Risk of bias	
Faramarzi M et al. (2017) [26]	○	○	?	○	○	○	○	low	
Phogat M et al. (2014) [27]	?	?	X	x	?	?	○	high	exclusion
Jain M et al. (2013) [28]	?	?	X	x	○	?	○	high	exclusion
Chitsazi MT et al. (2013) [29]	○	○	?	○	○	○	○	low	
Chauhan AS et al. (2013) [30]	?	?	X	x	○	?	○	high	exclusion
Verma A et al. (2012) [32]	?	?	X	x	?	?	○	high	exclusion
Matesanz P et al. (2013) [31]	○	○	○	○	○	○	○	low	
Kranti K et al. (2010) [33]	○	○	○	○	?	?	○	unclear	
Paolantonio M. (2009) [34]	○	?	X	○	○	○	○	high	
Gupta R et al. (2008) [35]	?	?	X	x	?	?	○	high	exclusion
Fonseca DC 2015 [17]	○	○	○	?	○	○	○	low	
santos VR 2013 [18]	○	○	○	?	○	○	○	low	
Quirynen M 2006 [2]	○	○	x	?	○	○	○	high	
Swierkot K 2009 [19]	○	○	?	x	○	○	○	high	
unsal E 1994 [11]	?	?	x	x	○	○	○	high	exclusion
lecic J 2016 [10]	○	○	?	○	○	○	○	low	
Oosterwaal PJM 1991 [12]	○	?	○	○	?	○	○	unclear	

○: low risk of bias;?: unclear risk of bias; x: high risk of bias;

Exclusion: when a trial did not meet all four criteria for randomization and blinding methods, it was excluded from quantitative analysis, as its low quality and high bias may have subverted the validity of the results and conclusions

### Synthesis of results

All 17 studies reported on clinical outcomes with the use of adjunctive CHX Gel. The clinical results of these studies are summarized in Additional file 2: Table S2. There was no consensus on the clinical efficacy of adjunctive CHX gel to SRP at selected sites. A significant improvement in PPD and/or CAL was reported in a number of studies using XAN-CHX gel [27, 28, 30, 32–35]. Whereas, several studies showed no additional benefit in clinical outcomes with the adjunctive use of CHX gel [10–12, 26, 29, 31]. In addition, all three studies using CHX gels that did not contain Xanthan gum reported no clinical benefits in the test group [10–12]. For comparing FMD and FMSRP, one study showed a significant improvement of PPD at 6 months [17]. In the other three studies, no sufficient evidence supported that FMD provided any significant improved clinical outcomes in terms of PPD and CAL [2, 18, 19].

Quantitative analysis was performed when data on at least three studies at 3 and/or 6 months follow-up (± 2 months) was obtained. Six trials were excluded because of an unreached methodological quality for the requirement of this meta-analysis. Four trials were not included in the quantitative synthesis due to a lack of clinical outcomes in terms of PPD and CAL at follow-up [2, 12, 33, 34]. Finally, four studies were included for the quantitative analysis of subgingival application of CHX gel at selected sites in terms of PPD reduction and CAL gain^10,26,29,31^, three studies were included for analysis of full-mouth subgingival application of CHX gel in terms of the mean PPD and mean CAL at 3–4 and 6–8 months [17–19]. Four trials reported the adverse events after treatment [18, 19, 29, 32]. Changes in PPD and CAL at selected sites 6 months after CHX gel administration and the mean bleeding of probing (BOP) value at follow-ups after treatment were not conducted due to a lack of data available in the meta-analysis.

### Pooled outcomes

**Fig. 2:**
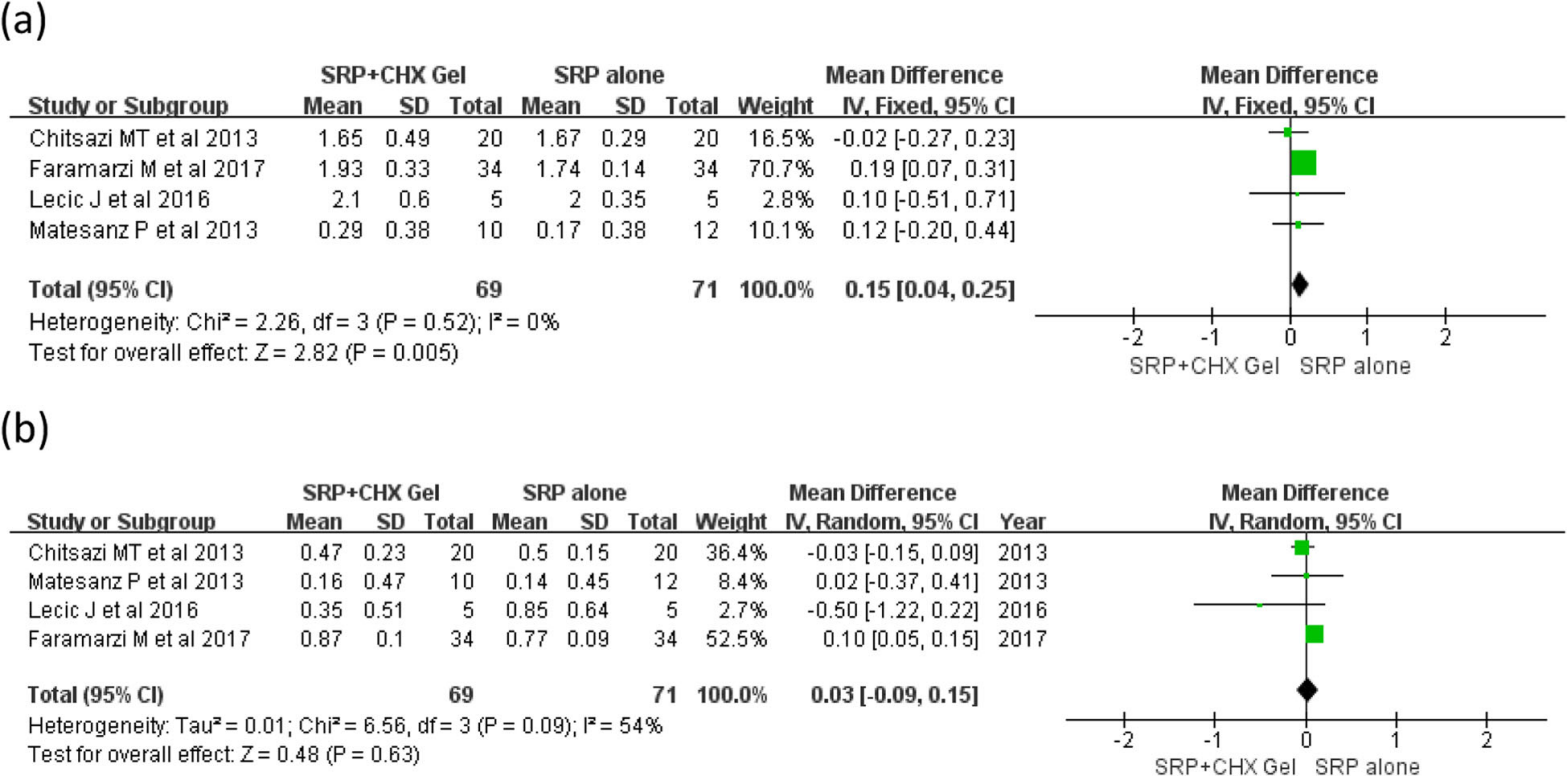
Forest plots comparing the adjunctive use of chlorhexidine (CHX) gel to scaling and root planing (SRP) and SRP alone at selected sites at 3 months: **a** probing pocket depth (PPD) reduction; **b** clinical attachment level (CAL) gain

**Fig. 3:**
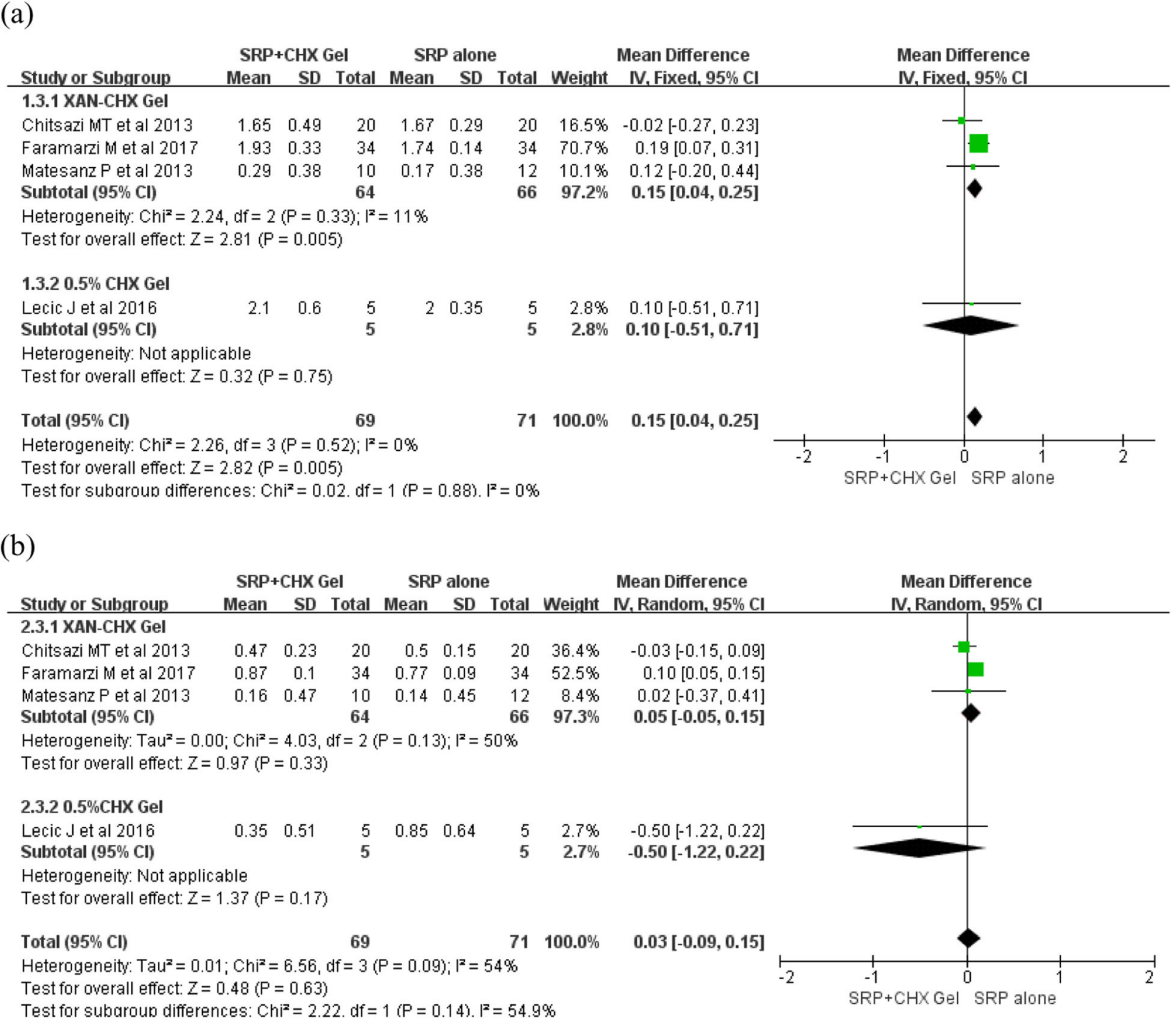
Forest plots for subgroup analysis of PPD reduction and the CAL gained between the adjunctive use of CHX gel to SRP and SRP alone at selected sites at 3 months: **a** PPD reduction; **b** CAL gain

For the adjunctive application of CHX gel to SRP compared to SRP alone at selected sites, the meta-analysis showed a significant improvement in PPD reduction, with a mean MD of 0.15 mm (MD: 0.15 [95% CI: 0.04–0.25]; *p* = 0.005), no heterogeneity was observed among the studies (I2 = 0%) (Fig. 2a); No significant differences were found on the CAL gain between the groups (MD: 0.03 [95% CI: − 0.09–0.15]; *p* = 0.09) and moderate heterogeneity was indicated (I2 = 54%) (Fig. 2b). For subgroup analysis, adjunctive XAN-CHX gel provided a significant PPD reduction, with a MD of 0.15 mm with no heterogeneity (MD: 0.15 [95% CI: 0.04–0.25]; *p* = 0.005, I2 = 11%) (Fig. 3a). Whereas, no additional benefit for CAL gain was showed in the XAN-CHX group with a low heterogeneity among the studies (MD: 0.05 [95% CI: − 0.05–0.15]; *p* = 0.33, I2 = 50%) (Fig. 3b).

**Fig. 4:**
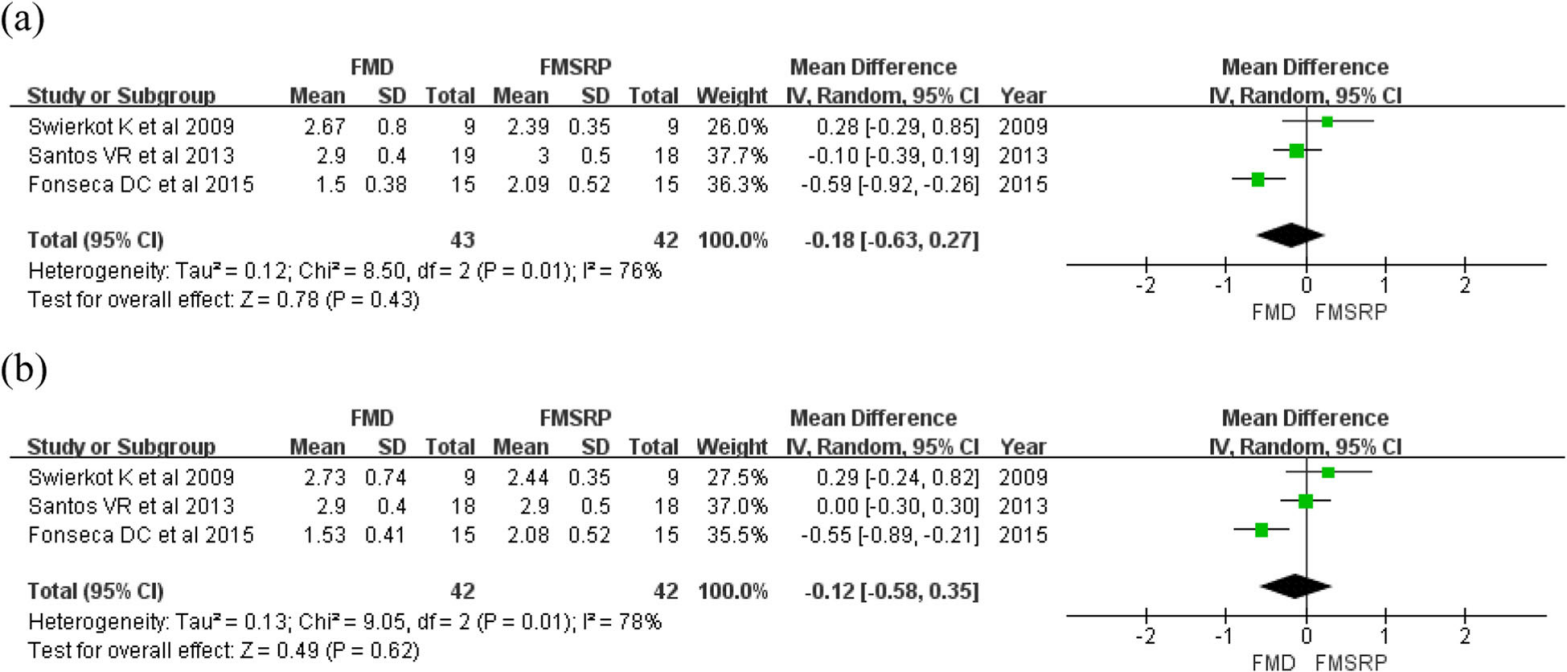
Forest plots of the mean PPD at 3 and 6 months comparing full-mouth disinfection (FMD) and full-mouth scaling and root planing (FMSRP): **a** at 3–4 months, **b** at 6–8 months

**Fig. 5:**
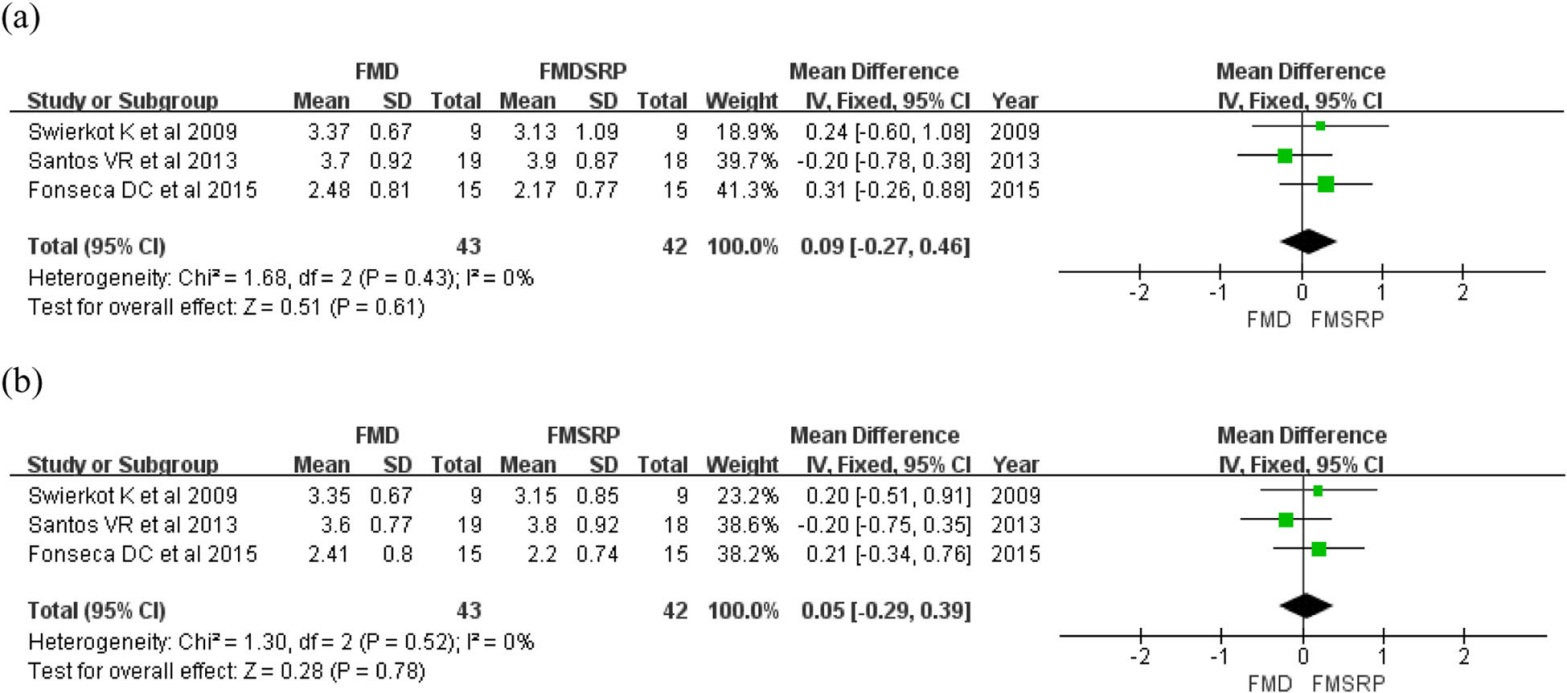
Forest plots of the mean CAL at 3 and 6 months comparing full-mouth disinfection (FMD) and full-mouth scaling and root planing (FMSRP): **a** at 3–4 months, **b** at 6–8 months

For full-mouth use of CHX gel, both the mean PPD and CAL showed no significant differences at 3–4 and 6–8 months. The overall effect size for PPD was − 0.18 mm at 3–4 months and − 0.12 mm at 6–8 months, and a high heterogeneity was observed among the studies [3–4 months (MD: -0.43 [95% CI: − 0.63–0.27]; *p* = 0.43, I2 = 76%) (Fig. 4a), 6–8 months (− 0.12 [95% CI: − 0.58–0.35]; *p* = 0.62, I2 = 78%) (Fig. 4b)]. CAL was 0.09 mm at 3–4 months and 0.05 mm at 6–8 months with no heterogeneity [3–4 months (MD: 0.09 [95% CI: − 0.27–0.46]; *p* = 0.61, I2 = 0%) (Fig. 5a), 6–8 months (MD: 0.05 [95% CI: − 0.29–0.39]; *p* = 0.78, I2 = 0%) (Fig. 5b)].

### Adverse events

Four studies reported adverse effects after treatment [17, 18, 28, 31]. Only one study comparing FMD and FMSRP reported that 17 subjects in the FMD and 12 in the FMSRP groups had one or two adverse events following mouth rinses, including changes in taste perception, dry mouth and staining [17].

## Discussion

Four trials comparing adjunctive CHX gel and SRP with SRP alone at selected sites were included for quantitative analysis. The results showed that adjunctive administration of CHX gel provided a significant improvement in PPD reduction with a small overall effect size of 0.15 mm and no benefit to CAL. For subgroup analysis, adjunctive subgingival administration of XAN-CHX gel containing 1.5% CHX provided also a slightly greater improvement of PPD reduction of MD 0.15 mm.

In qualitative analysis, CHX gel without Xanthan gum was applied as adjunct to SRP at selected sites in three studies, and showed no beneficial clinical outcomes [10–12]. The results were consistent with various studies reporting minimal benefits in the local use of traditional CHX gel as a monotherapy [36, 37] or as an adjunct to SRP [10–13]. The effect of locally delivered antimicrobial drugs depends on its concentration and contact time in the local environment [12]. For subgingival administration of drugs, the outflow of crevicular fluid may play an important role. Evidence indicated that the outflow of crevicular fluid is about 20 ml/hour, which might be the main cause of the short-term half-life of the gel within the periodontal pocket [14, 38]. Oosterwaal et al. applied fluorescein gel in four pockets of 10 patients, samples were taken from 1 of the 4 pockets at 5, 10, 20 and 40 min. The results showed that the most locally delivered gels in the pocket disappeared within 5 min after application, which might be due to the elasticity of pocket soft tissue, bleeding after drug administration and spreading of the gel. And then the gel was washed out linearly and gradually by crevicular fluid flow and released from the adherent surface of the periodontal pocket [14]. Given the high clearance of CHX within the pockets, CHX gel seemed to not be an effective adjuvant to SRP. XAN-CHX gel has been applied for local periodontal treatment within the recent 10 years, which contains a mixture of CHX digluconate and CHX dihydrochloride, incorporated in a Xanthan gum. XAN-CHX gel demonstrated a greater capacity to increase viscosity of the carrier (CHX) and maintained the bacteriostatic and bactericidal concentrations for at least 2 weeks inside the periodontal pocket [35], which could further promote its pharmacotherapeutic effects. Based on this evidence, XAN-CHX gel may overcome the limitations of the previously used CHX gel. Considering the results of this meta-analysis for subgroups, XAN-CHX gel provided only a minor additional improvement with mean MD of 0.15 mm of PPD reduction, and no benefit of CAL gain. So far, no sufficient data have supported the clinical efficacy of adjunctive subgingival applications of XAN-CHX gel according to existing research. Evidence has reported that CHX has a high affinity for salivary or serum proteins and blood, which might lead to its rapid concentration decrease in the subgingival environment [39–41]. Furthermore, the behaviour of pathogenic bacteria in periodontal pocket may also resist the effectiveness of local CHX gel. Evidence supported that P.g releases vesicles capable of inactivating the CHX molecule, thereby protecting themselves and other bacteria from the bactericidal activity [15]. These features may markedly negatively regulate the effects of subgingival administrations of a XAN-CHX gel. Microbiological outcomes of various studies have confirmed the minimal efficacy of locally delivered XAN-CHX gel as an adjunct to SRP, which showed minor bacterial count reductions in an adjunctive XAN-CHX gel group as compared to control [29, 31, 34, 42]. Due to a lack of microbiological data from consistent testing methods and standards, microbiological outcomes were not analysed in the review.

In recent years, CHX gel has been commonly used for FMD protocol in the treatment of periodontal disease. Considering the clinical benefit of FMD in varying degrees, FMD protocol has been conducted in a large number of studies [43–46]. In addition to full-mouth subgingival applications of CHX gel, tongue brush with CHX gel and mouthwash with CHX solution were also performed with the aim of maximum elimination of periodontal pathogens in the mouth. Despite these, no additional benefits for the adjunctive use of CHX in FMSRP were shown in this meta-analysis. This result is consistent with other studies and reviews, which indicated that the benefits of FMD probably resulted from the short-term full-mouth mechanical debridement, rather than the beneficial effects of CHX [15, 47]. A high heterogeneity was detected for analysis of the mean PPD between the FMD and FMSRP groups at 3 months (I2 = 76%) and 6 months (I2 = 78%). Regarding the small number of included studies and limited data available, there were variable factors impacting on the results, such as the general health of the included patients, the initial disease severity of the chronic periodontitis, the frequency of CHX gel application and the influence of other adjunctive means of CHX included in FMD and its period and frequency.

Noticeably, side effects and adverse events related to the use of the local administration of CHX in the treatment of periodontitis should be taken into account and be weighed against the potential benefits. Although the local application of antiseptics or antibiotics overcomes uncertainties in the systemic use of antibiotics, adverse events, such as changes in taste perception, dry mouth, erythema, oral ulceration, gingival tingling, periodontal abscesses, root sensitivity and staining of tongue or teeth, were reported [48]. For FMD, due to long-term mouthwash using CHX solution, staining could occur in most patients [18]. This fact should remind clinicians that the balance between the small effect size of clinical benefit and high possibility of tooth staining should be taken in consideration when developing a treatment plan for periodontal patients.

### Limitations

To the best of our knowledge, this is the first systematic review focusing on the effects of adjunctive subgingival application of CHX gel to SRP. Therefore, we cannot compare our results with previous publications. In addition, because of the lack of RCTs with high quality, only seven studies with a small number of participants were included for quantitative meta-analysis, and long-term clinical results comparing the test and control groups were not calculated. More RCTs with more participants and long-term follow-up are needed in the future.

## Conclusion

Based on the results of this meta-analysis, adjunctive subgingival administration of XAN-CHX gel at individual selected sites with PPD at least 4 mm promotes a slight additional benefit in PPD reduction. Adjunctive antiseptics of CHX gel at specific sites might be advisable, but SRP always plays the dominate role in the treatment of chronic periodontitis. Due to a lack of high-quality studies, more RCTs with larger sample sizes and strict standards are needed to confirm these conclusions.

## Supplementary information

**Additional file 1: Table S1.** Reasons for exclusion of studies.

**Additional file 2: Table S2.** Summary of clinical outcomes.

## Data Availability

The datasets used and/or analysed during the current study are available from the corresponding author on reasonable request.

## Abbreviations

CAL: Clinical attachment levels
CHX: Chlorhexidine
CP: Chronic periodontitis
FMD: Full-mouth disinfection
FMSRP: Full-mouth scaling and root planning
NSPT: Nonsurgical periodontal treatment
PPD: Probing pocket depth
SRP: Scaling and root planing
XAN-CHX: Xanthan-based chlorhexidine
