# Supplementary material for: Adjunctive subgingival application of Chlorhexidine gel in nonsurgical periodontal treatment for chronic periodontitis: a systematic review and meta-analysis
Source: BMC Oral Health. 2020 Jan 31;20:34. doi: 10.1186/s12903-020-1021-0 (PMC6995104; doi:10.1186/s12903-020-1021-0)
Supplement: Supplementary file 1 — Additional file 1: Table S1. Reasons for exclusion of studies. [file 12903_2020_1021_MOESM1_ESM.docx]

|  | **Supplemental Table 1. Reasons for exclusion of studies** | | |
| --- | --- | --- | --- |
|  | **STUDY** | **AUTHOR** | **REASON FOR EXCLUSION** |
| 1 | Comparative evaluation of subgingivally delivered chlorhexidine varnish and chlorhexidine gel in reducing microbial count after mechanical periodontal therapy | Manthena S et al (2015) | only microbioloical outcomes |
| 2 | Comparative evaluation of single application of 2% whole turmeric gel versus 1% chlorhexidine gel in chronic periodontitis patients: A pilot study. | Jaswal R et al (2014) | Follow-up<3 months |
| 3 | [Oral antiseptic and periodontitis: a clinical and microbiological study.](https://cghsrpa.cgmh.org.tw:30012/pubmed/25284540) | Mummolo S et al (2014) | not randomized |
| 4 | Short-Term Results in Evaluating a Gingiva-Adhesive Hydrophobic-Chlorhexidine-Gel for Chronic Periodontitis. | Sarbu C et al (2014) | no control group |
| 5 | Effect of Topical Gel Chlorhexidine 0.2% on Non-Surgical Treatment of Chronic Periodontitis | Vadiati Saberi B et al | not randomized |
| 6 | The rôle of chlorhexidine in the one-stage full-mouth disinfection treatment of patients with advanced adult periodontitis. Long-term clinical and microbiological observations. | Quirynen M et al (2000) | not randomized |
| 7 | Evaluation of a hydrophobic gel adhering to the gingiva in comparison with a standard water-soluble 1% chlorhexidine gel after scaling and root planing in patients with moderate chronic periodontitis. A randomized clinical trial. | Rusu D et al (2017) | not subgingival application |
| 8 | Clinical and microbiological benefits of strict supragingival plaque control as part of the active phase of periodontal therapy. | Feres M et al (2009) | not CHX gel |
| 9 | The antimicrobial and clinical effects of a single subgingival irrigation of chlorhexidine in advanced periodontal lesions. | Lander PE et al (1986) | monotherapy of CHX gel |
| 10 | Subgingival utilization of a 1% chlorhexidine collagen gel for the treatment of periodontal pockets. A clinical and microbiological study. | Vinholis AH et al (2001) | not randomized |
