# Supplementary material for: Adjunctive subgingival application of Chlorhexidine gel in nonsurgical periodontal treatment for chronic periodontitis: a systematic review and meta-analysis
Source: BMC Oral Health. 2020 Jan 31;20:34. doi: 10.1186/s12903-020-1021-0 (PMC6995104; doi:10.1186/s12903-020-1021-0)
Supplement: Supplementary file 2 — Additional file 2: Table S2. Summary of clinical outcomes. [file 12903_2020_1021_MOESM2_ESM.docx]

| **Supplemental Table 2. Summary of clinical outcomes** | | | | | | | |
| --- | --- | --- | --- | --- | --- | --- | --- |
| **clinical outcomes comparing adjunctive usd of CHX Gel to SRP and SRP alone/placebo at selected sites** | | | | | | | |
| Study |  | **PPD (mm, Mean values ± SD)** | | | **CAL (mm, Mean values ± SD)** | | |
|  |  | baseline | 3 month | PPD reduction | baseline | 3 month | CAL gain |
| Faramarzi M et al. (2017) | Control | 5.41±0.76 | 3.67±0.83 | 1.74±0.14 | 4.67±0.54 | 3.90±0.74 | 0.77±0.09 |
|  | Test | 5.41±0.80 | 3.48±0.56 | 1.93±0.33 | 5.06±0.67 | 4.19±0.79 | 0.87±0.10 |
| Chauhan AS et al. (2013) | Control | 5.90±0.27 | 4.30±0.33 | 1.60±0.27 | 6.10±0.38 | 5.55±0.37 | 0.55±0.16 |
|  | Test | 5.95±0.31 | 3.48±0.34 | 2.48±0.32 | 6.15±0.36 | 5.03±0.36 | 1.13±0.27 |
| Phogat M et al. (2014) | Control | / | / | 2.264±0.031 | / | / | 2.405±0.079 |
|  | Test | / | / | 3.764±0.01 | / | / | 2.913±0.051 |
| Matesanz P et al. (2013) | Control | 3.73±0.45 | 3.54±0.45 | 0.17±0.38 | 4.72±1.25 | 4.6±1.25 | 0.14±0.45 |
|  | Test | 3.58±0.47 | 3.32±0.47 | 0.29±0.38 | 4.31±0.98 | 4.12±0.98 | 0.16±0.47 |
| Chitsazi MT et al. (2013) | Control | 4.9±0.78 | 3.25±0.65 | 1.67 | 3.9±0.58 | 3.4±0.60 | 0.5 |
|  | Test | 5.05±0.75 | 3.38±0.79 | 1.65 | 4.15±0.67 | 3.67±0.65 | 0.47 |
| Jain M et al. (2013) | Control | 5.20±0.484 | 3.07±0.691 | / | 11.43±2.750 | 9.20±2.845 | / |
|  | Test | 5.20±0.484 | 2.50±0.731 | / | 11.70±2.806 | 10.03±2.977 | / |
| Verma A et al. (2012) | Control | 6.39±0.95 | 5.17±1.12 | / | 6.65±1.12 | 5.76±1.21 | / |
|  | Test | 6.41±0.96 | 4.26±1.00 | / | 6.70±1.21 | 5.17±1.18 | / |
| Kranti K et al (2010) | Control | Data not available | | | | | |
|  | Test |  |  |  |  |  |  |
| Paolantonio M. (2009) | Control | Data not available | | | | | |
|  | Test |  |  |  |  |  |  |
| Gupta R et al. (2008) | Control | 6.03±1.15 | 4.30±0.87 | 1.73±0.94 | 6.00±1.11 | 5.13±0.86 | 0.86±0.68 |
|  | Test | 6.40±0.89 | 3.63±1.29 | 2.76±1.25 | 6.53±1.00 | 4.50±1.30 | 2.03±1.22 |
| lecic J et al. (2016) | Control | 5.25±0.55 | 3.25±0.55 | / | 4.05±1.35 | 3.20±0.76 | / |
|  | Test | 5.05±1.00 | 2.95±0.75 | / | 3.75±1.20 | 3.40± 0.82 | / |
| unsal E et al. (1994) | Control | 5,14±1,45 | 3.31±0.73 | 1.83±0.54 | 3.66±1.22 | 2.62±1.24 | 1.04±0.16 |
|  | Test | 4,90±1.11 | 3.32±1.01 | 1.58±0.96 | 4,03±1,50 | 3.33±1.76 | 0.70±1.09 |
| Oosterwaal PJM et al. (1991) | Control | Data not available | | | | | |
|  | Test |  |  |  |  |  |  |
| **clinical outcomes comparing FMD and FMSRP** | | | | | | | |
| Study | **PPD (mm, Mean values ± SD)** | | | | **CAL (mm, Mean values ± SD)** | | |
|  |  | baseline | 3 months | 6 months | baseline | 3 months | 6 months |
| Fonseca DC et al. (2015) | Control | 2.27±0.60 | 2.09±0.52 | 2.08±0.52 | 2.39±0.99 | 2.17±0.77 | 2.20±0.74 |
|  | Test | 2.10±0.50 | 1.50±0.38 | 1.53±0.41 | 2.84±0.95 | 2.48±0.81 | 2.41±0.80 |
| santos VR et al. (2013) | Control | 3.7±0.8 | 3.0±0.5 | 2.9±0.5 | 4.4±0.99 | 3.9±0.87 | 3.8±0.92 |
|  | Test | 3.4±0.5 | 2.9±0.4 | 2.9±0.4 | 4.1±0.85 | 3.7±0.92 | 3.6±0.77 |
|  |  | baseline | 4 months | 8 months | baseline | 4 months | 8 months |
| Swierkot K et al. (2009) | Control | 3.20±0.57 | 2.39±0.35 | 2.44±0.35 | 3.68±0.93 | 3.13±1.09 | 3.15±0.85 |
|  | Test | 3.55±0.88 | 2.67±0.80 | 2.73±0.74 | 4.20±0.69 | 3.37±0.67 | 3.35±0.67 |
| Quirynen M et al. (2006) | Control | singel rooted: medium pocket: 4.9±0.3 moderate pocket: 6.8±0.3 Multi-rooted: medium pocket: 5.2±0.2 moderate pocket:7.3±0.5 | / | singel rooted: medium pocket: 3.5±0.5 moderate pocket: 4.3±0.8 Multi-rooted: medium pocket:3.7±0.4 moderate pocket: 4.7±0.7 | Data not available | | |
|  | Test | Single rooted: medium pocket: 4.9±0.2 moderate pocket: 6.9±0.5 Multi-rooted: medium pocket: 4.9±0.4 moderate pocket: 7.3±0.6 | / | Single rooted: medium pocket: 3.1±0.4 moderate pocket:4.3±0.7 Multi-rooted: medium pocket: 3.4±0.7 moderate pocket: 4.6±0.7 |  |  |  |
